# Percutaneous autologous impaction bone graft for advanced femoral head osteonecrosis: a retrospective observational study of unsatisfactory short-term outcomes

**DOI:** 10.1186/s13018-021-02288-7

**Published:** 2021-02-17

**Authors:** Yutaka Kuroda, Manabu Nankaku, Yaichiro Okuzu, Toshiyuki Kawai, Koji Goto, Shuichi Matsuda

**Affiliations:** 1 Department of Orthopaedic Surgery, Graduate School of Medicine, Kyoto University, Shogoin, Kawahara-cho 54, Sakyo-ku, Kyoto, 606-8507 Japan; 2 Rehabilitation Unit, Kyoto University Hospital, Kyoto, Japan

**Keywords:** Osteonecrosis, Femoral head, Avascular necrosis, Joint-preserving surgery, Bone graft, Core decompression, Total hip arthroplasty, Collapse, Survivorship, Regenerative therapy

## Abstract

**Background:**

Half of osteonecrosis of the femoral head (ONFH) patients suffer femoral head collapse at initial diagnosis, and more than half are bilaterally affected. This study developed a percutaneous autologous impaction bone graft (IBG) technique as a modification of core decompression (CD). We also summarized the short-term results and treatment efficacy of percutaneous autologous IBG in advanced ONFH.

**Methods:**

Twenty patients (12 males, 8 females) with nontraumatic, postcollapse ONFH except one case underwent CD (10-mm core diameter) and reverse IBG. Radiological changes of the ONFH stage and type were analyzed. Survival analysis using Kaplan–Meier estimates was performed with conversion to total hip arthroplasty (THA) as the endpoint. In addition, the Harris hip score (HHS) and University of California, Los Angeles (UCLA) activity rating scale were evaluated.

**Results:**

Percutaneous autologous IBG was performed successfully, with an average operation time of < 1 h and small blood loss, and 7 patients (35%) needed conversion to THA at an average of 17 months postoperatively. We observed radiological progressive change in 60% of the patients during a mean observation period of 3 years. The mean clinical scores, except data recorded, after THA significantly improved (before vs. after 3 years: UCLA activity score, 3.7 vs. 5.2 [*P* = 0.014]; HHS, 57.6 vs. 76.5 points [*P* = 0.005]). In addition, 6 patients showed radiological progression but no clinical deterioration.

**Conclusions:**

Percutaneous autologous IBG was technically simple and minimally invasive, but short-term results were unsatisfactory for advanced ONFH. Indications for this procedure should be carefully examined to improve it in order to enable bone formation.

## Background

Osteonecrosis of the femoral head (ONFH) is a multifactorial disease with unclear pathogenesis. It occurs at ~ 30 years of age and is the main reason for total hip arthroplasty (THA) in young patients. Patients undergoing systemic corticosteroid pulse therapy and people who abuse alcohol are at risk of ONFH [1–5]. The use of magnetic resonance imaging (MRI) helps in diagnosing ONFH at an early stage [1–3, 5, 6]. In the USA, the number of surgical procedures for ONFH in 2008 was double that in 1992 [7]. Because the long-term outcomes of THA for ONFH have improved [2–4, 8], THA is selected as the first, not the final, surgical option [7]. However, ONFH patients are young, and THA is not an easy choice because of the risks of nondurability, infection, and dislocation of artificial implants [2]. Historically, in Europe and the USA, bone grafts and core decompression (CD) were performed to preserve joints [1–7], while in Japan and the rest of Asia, osteotomy was used [1–6, 9]. In the 1940s, Phemister et al. [10] performed tibial bone graft from the lateral femur to the femoral head via a core, which has evolved over time [1–6]. Since the 1960s, CD has also become popular [1–7, 11]. With increasing use of THA, the development of regenerative medicine combining CD with cell transplantation, bone substitute, and growth factors has shown promise, especially for early to stage 2 ONFH, prior to femoral head collapse [1, 2, 4–6], with THA being the final surgical option for ONFH. However, half of advanced (stage 3 or greater) ONFH patients already have femoral head collapse at initial diagnosis [12], and more than half are bilaterally affected [1, 5, 12]. Therefore, it is necessary to establish joint-preserving surgery for advanced ONFH. This study developed a percutaneous autologous impaction bone graft (IBG) technique as a modification of CD and the Phemister method. The femoral bone obtained from CD was itself used as a reverse autologous bone graft. We also summarized the short-term (3-year) results and treatment efficacy of percutaneous autologous IBG in advanced ONFH.

## Methods

### Ethics

This retrospective observational study was approved by the institutional review board of Kyoto University Graduate School and Faculty of Medicine. The study was conducted in accordance with the World Medical Association Declaration of Helsinki. All patients provided written informed consent for the procedure and publication of study results.

### Patients

We enrolled 20 patients (20 hip joints; 12 men and 8 women; mean age, 41 years [range 18–70 years]) with advanced ONFH except one case in this study. The inclusion criteria was nontraumatic ONFH patients (stage 2 and stage 3) who requested and accepted for the joint-preserving surgery. The key exclusion criteria were traumatic ONFH and patients with stage 4 diagnosed by magnetic resonance imaging (MRI) in accordance with the 2001 Japanese Investigation Committee (JIC) guidelines [13]. Stage 3 shows radiological femoral head collapse but no acetabular deformity. Of the 20 patients, 12 were associated with corticosteroid use, 5 were associated alcohol intake, 2 were associated corticosteroid use and alcohol intake, and 1 was idiopathic. As for JIC classification, 1 had stage 2, 17 had stage 3A, and 2 had stage 3B ONFH; 2 patients had type C1 and 18 had type C2 lesions; and 10 patients had bilateral morbidity and 3 had already undergone THA. Table 1 shows patient demographics at the baseline, along with key results 3 years postoperatively.

**Table 1:** Patient baseline characteristics, surgical overview, and status at final follow-up (*n* = 20)

Case	Age (years)/sex	Background factors for ONFH (disease)	Side	Baseline stage/type	Opposite side stage/type	Surgery time (min)/blood loss (g)	Stage/type at recent follow-up	Follow-up period (months)
1	24/F	Steroid use (MCTD)	R	Stage 3A/type C2	Healthy	122/80	Stage 3A/type C2	42
2	53/F	Steroid use (dermatomyositis)	R	Stage 3A/type C2	rhFGF-2 therapy^a^ Stage 1/type C2	96/35	THA	42
3	41/M	Alcohol intake	R	Stage 3A/type C2	Healthy	60/180	THA	42
4	33/M	Idiopathic	R	Stage 3B/type C2	Healthy	50/10	Stage 3B/type C2	40
5	29/F	Steroid use (Alopecia areata)	L	rhFGF-2 therapy^a^ Stage 3A/type C2	THA^b^	67/10	Stage 4/type C2	40
6	18/F	Steroid use (ALL)	R	Stage 3B/type C2	Stage 3A/type C2	48/60	Stage 3B/type C2	39
7	56/M	Steroid use (IP) + alcohol intake	R	Stage 3A/type C2	THA^b^	50/350^c^	Stage 3B/type C2	38
8	43/M	Steroid use (EG)	R	Stage 3A/type C2	rhFGF-2 therapy^a^ Stage 1/type C2	42/10	THA	38
9	41/M	Steroid use (EPGA)	L	Stage 3A/type C2	Healthy	67/10	THA	37
10	36/F	Steroid use (MS)	L	Stage 3A/type C1	Healthy	39/10	THA	37
11	43/M	Alcohol intake	R	Stage 3A/type C2	Healthy	46/10	Stage 3A/type C2	36
12	49/M	Steroid use (IP) + Alcohol intake	L	Stage 3A/type C2	Healthy	38/10	Stage 3B/type C2	36
13	45/M	Steroid use (nephrotic synd.)	R	Stage 3A/type C2	rhFGF-2 therapy^a^ Stage 3A/type C2	60/10	Stage 3A/type C2	34
14	27/F	Steroid use (SLE)	R	Stage 3A/type C2	Healthy	40/10	Stage 3B/type C2	34
15	45/M	Steroid use (SS, IP)	R	Stage 3A/type C2	Healthy	38/10	Stage 3A/type C2	33
16	44/M	Steroid use (Bullous)	R	Stage 2/type C1	Bone grafting Stage 2/type C1	50/10	Stage 2/type C1	33
17	44/F	Alcohol intake	R	Stage 3A/type C2	THA	62/10	Stage 3B/type C2	32
18	36/M	Alcohol intake	R	Stage 3A/type C2	Healthy	30/10	Stage 3A/type C2	32
19	44/M	Alcohol intake	L	Stage 3A/type C2	Stage 1/type C2	47/10	THA	31
20	70/F	Steroid use (nephrotic synd.)	L	Stage 3A/type C2	Stage 2/type C1	39/10	THA	29
	Mean ± SD, 41±11; median (range), 43 (18–70); M:F=12:8	Steroid use, 12; alcohol intake, 5; idiopathic, 1; steroid use + alcohol use, 2	R, 12; L, 8	Stage 2 = 1; stage 3A = 17; stage 3B = 2/type C1, 2; type C2, 18	THA = 3; healthy = 10; stage 1 = 3; stage 2 = 2; stage 3A = 2/type C1, 2; type C2, 5	Mean ± SD 54±21/42±83; median (range), 49 (30–122)/10 (10–350)	THA = 7; stage 2 = 1; stage 3A = 5; stage 3B = 6; stage 4 = 1/type C1, 1; type C2, 12	Mean ± SD 36±3; median (range), 36.5 (29–42)

Type A had the smallest osteonecrosis, while type C2 had the largest osteonecrosis extended laterally to the acetabular edge. Stage 3A: femoral head collapse within 3 mm. Underline: detected radiological progression (radiological progressive change classified by the Japanese investigation committee classification). Underline: the operated side

*M* male, *F* female, *SD* standard deviation, *ONFH* osteonecrosis of the femoral head, *MCTD* mixed connective tissue disease, *ALL* acute lymphocytic leukemia, *IP* interstitial pneumonia, *EG* eosinophilic gastroenteritis, *EPGA* eosinophilic granulomatosis with polyangiitis, *MS* multiple sclerosis, *synd* syndrome, *SLE* systemic lupus erythematosus, *SS* Sjögren’s syndrome, *R* right, *L* left, *rhFGF* recombinant human fibroblast growth factor, *THA* total hip arthroplasty

^a^Clinical trial of rhFGF-2 regenerative therapy was previously performed in our hospital

^b^Both ONFH sides were operated simultaneously. THA was performed first, followed by bone grafting on the opposite side using the collapsed femoral head removed during THA

^c^The total volume loss including THA on the opposite side

### Surgical procedure

**Fig. 1:**
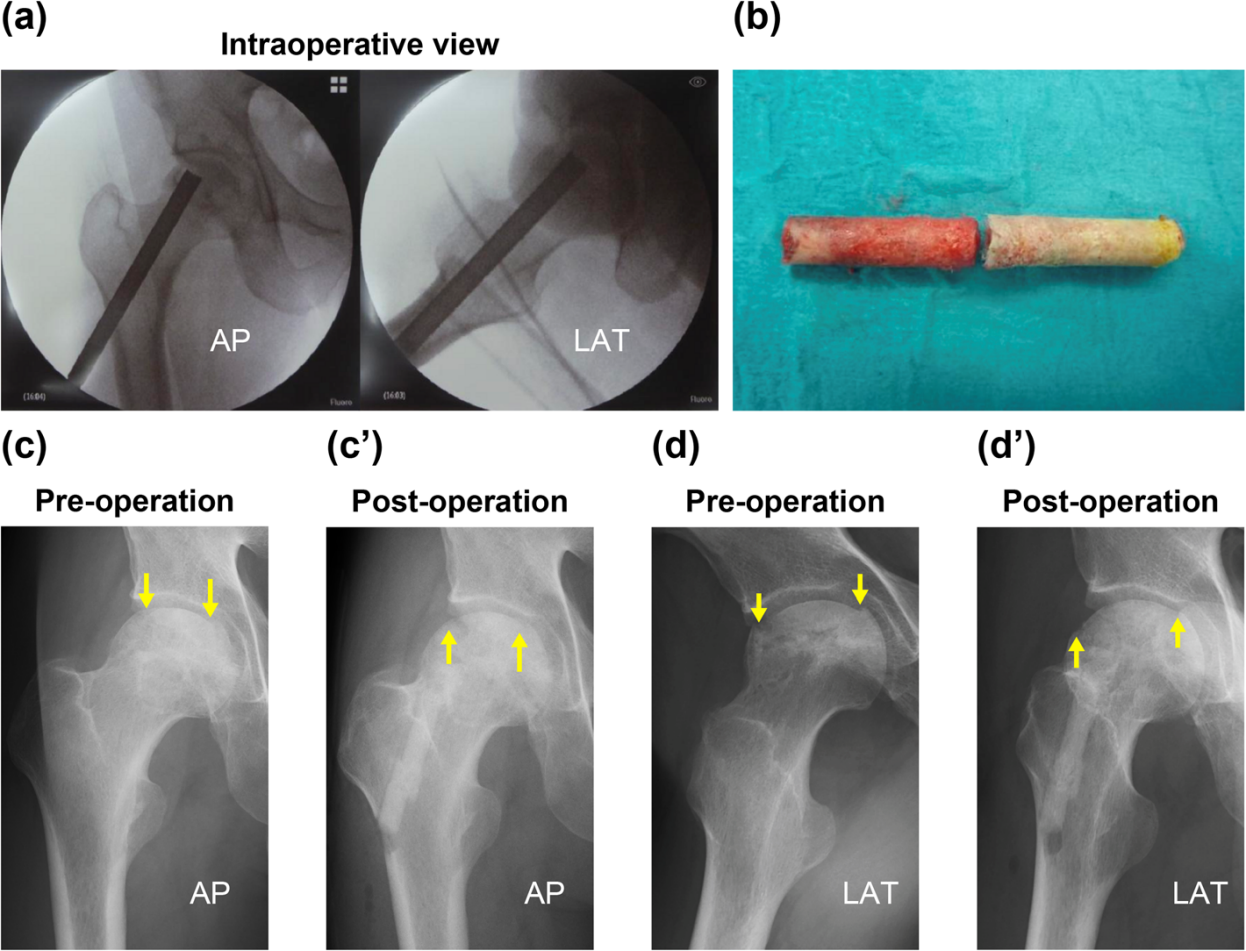
Surgical procedure, as shown in pre- and postoperative radiographs and intraoperative photographs. **a** Representative intraoperative fluoroscopic image at CD. **b** Photograph of a cylindrical bone taken by CD. The bloody zone is healthy, and the yellowish-white part on the right is the osteonecrotic part. Reverse IBG was performed. Postoperatively, the gap was repaired. Core defects in the healthy zone were supplemented with artificial bone avoiding fracture. **c**, **c’** Changes on AP X-ray. **d**, **d’** Changes on LAT X-ray. Downward yellow arrows indicate the gap made by femoral head collapse, while the upward yellow arrow indicates repair of the femoral head sphericity by IBG. CD, core decompression; IBG, impaction bone graft; AP, anteroposterior; LAT, lateral

We performed percutaneous autologous IBG on the 20 patients from December 2016 to January 2018. Each patient was anesthetized and placed in the supine position on a fracture operating table equipped with a C-arm image intensifier. A 3–5-cm longitudinal skin incision was made distal to the lateral aspect of the femur near the level of the lesser trochanter, and the femoral fascia and vastus lateralis muscle were split to reach the lateral part of the femoral cortex, the CD entry site. The approach was very similar to inserting a lag screw in γ-nail surgery for femoral neck fracture. On the basis of preoperative planning, using C-arm images, CD was carefully directed toward the center of the ONFH area. Next, using a soft-tissue protector, a 10-mm-diameter trephine was inserted from the femoral neck to the ONFH target site while checking two directions of fluoroscopy. Then, the trephine was pulled out, and the cylindrical bone inside it removed. IBG was performed using this cylindrical autologous bone in the opposite direction so that the healthy part was implanted in the ONFH area; fluoroscopy was performed to reconstruct the femoral head collapse (Fig. 1). For bone defects post-CD, we used two types of cylindrical artificial bones: 9-mm cylindrical hard hydroxyapatite (Neobone®, Aimedic MMT Co., Ltd., Tokyo, Japan) and 10-mm cylindrical unidirectional porous β-tricalcium phosphate (Affinos®, Kuraray Co., Ltd., Tokyo, Japan). The former artificial bone was used for the CD entry site, and the latter artificial bone was used for the proximal part of the femur.

In all 20 patients, surgery was performed as scheduled, with no problematic complications such as intraoperative fractures or postoperative infection. In 2 patients (cases 5 and 7), simultaneous bilateral surgeries were performed.

### Rehabilitation

Postoperative rehabilitation was performed under the guidance of a physical therapist with non-weight-bearing gait training from day 1 to 3 weeks postoperatively. Postoperative gait training included one-third partial weight bearing (PWB) at 3 weeks, half PWB at 4 weeks, two-thirds PWB at 5 weeks, and full weight bearing at 6 weeks postoperatively.

### Clinical assessment

We monitored perioperative complications related to surgery and assessed two clinical scores at the baseline and every 6 months postoperatively: the University of California, Los Angeles (UCLA) activity rating scale (1 [no physical activity] to 10 [regular participation in impact sports]) [14] and the Harris hip score (HHS) (0–100.025 points, with higher scores indicating better outcomes) [15]. The physical therapist assessed the patients’ walking capability, including passive hip range of motion as an HHS subcategory. We also performed survival analysis using the Kaplan–Meier method with conversion to THA as the endpoint.

### Radiographic assessment

We performed radiological evaluation based on the JIC ONFH classification [13]. At the time of the patients’ visit and every 6 months postoperatively, we took X-rays in two directions of the bilateral hip joint at the baseline. Computed tomography (CT) and MRI were also performed at the baseline. In addition, we evaluated a change in the JIC stage (1, 2, 3A, 3B, 4: early to secondary osteoarthritis via femoral head collapse) and JIC type (A, B, C1, C2: small to large) by X-ray.

### Statistical analysis

All statistical analyses were performed using JMP Pro 14 (SAS Institute, Cary, NC, USA). Clinical scores were analyzed using a repeated-measures linear mixed effects model [16]. The cumulative survival rate with THA as the endpoint was evaluated using the Kaplan–Meier method. Scores measured after THA conversion were excluded from clinical score analysis. *P* < 0.05 was considered statistically significant.

## Results

**Fig. 2:**
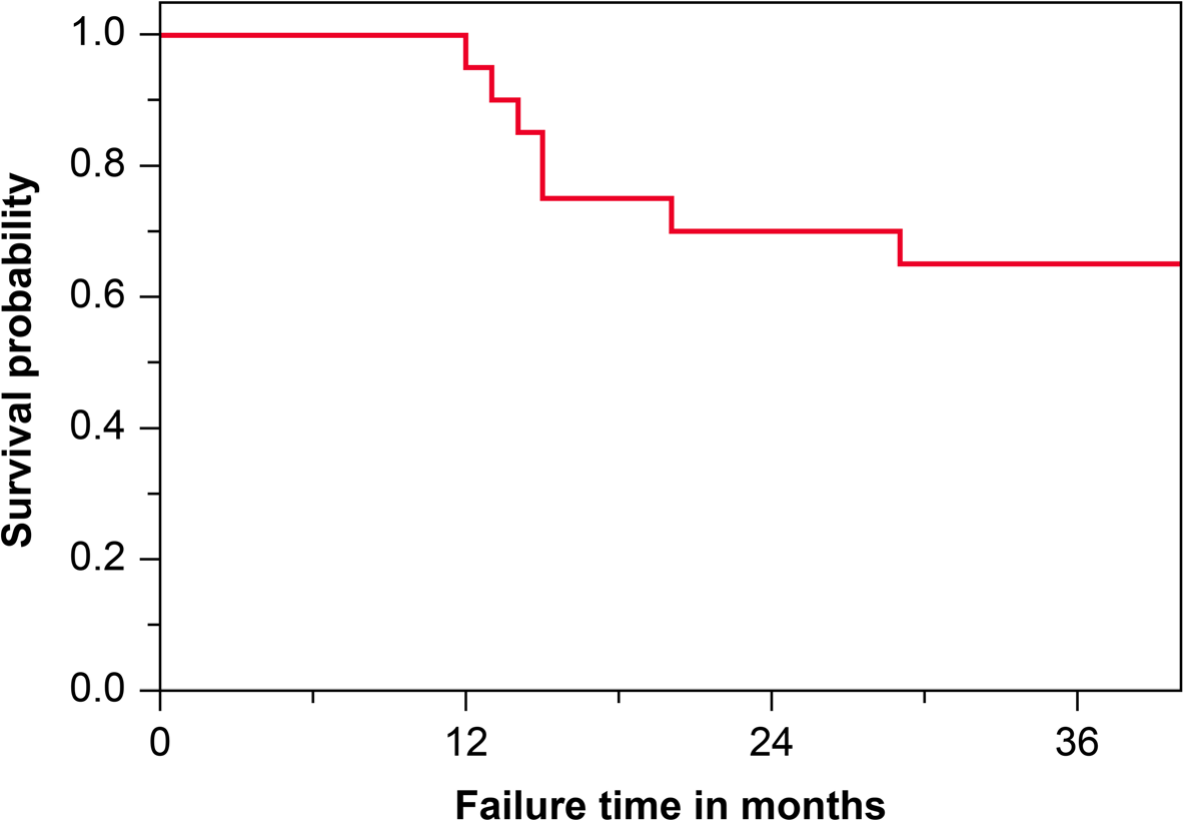
Radiological joint preservation by Kaplan–Meier survival analysis. The Kaplan–Meier estimates conversion to THA as the endpoint. The 3-year cumulative survival rate indicated that conversion to THA was 35%. THA, total hip arthroplasty

**Fig. 3:**
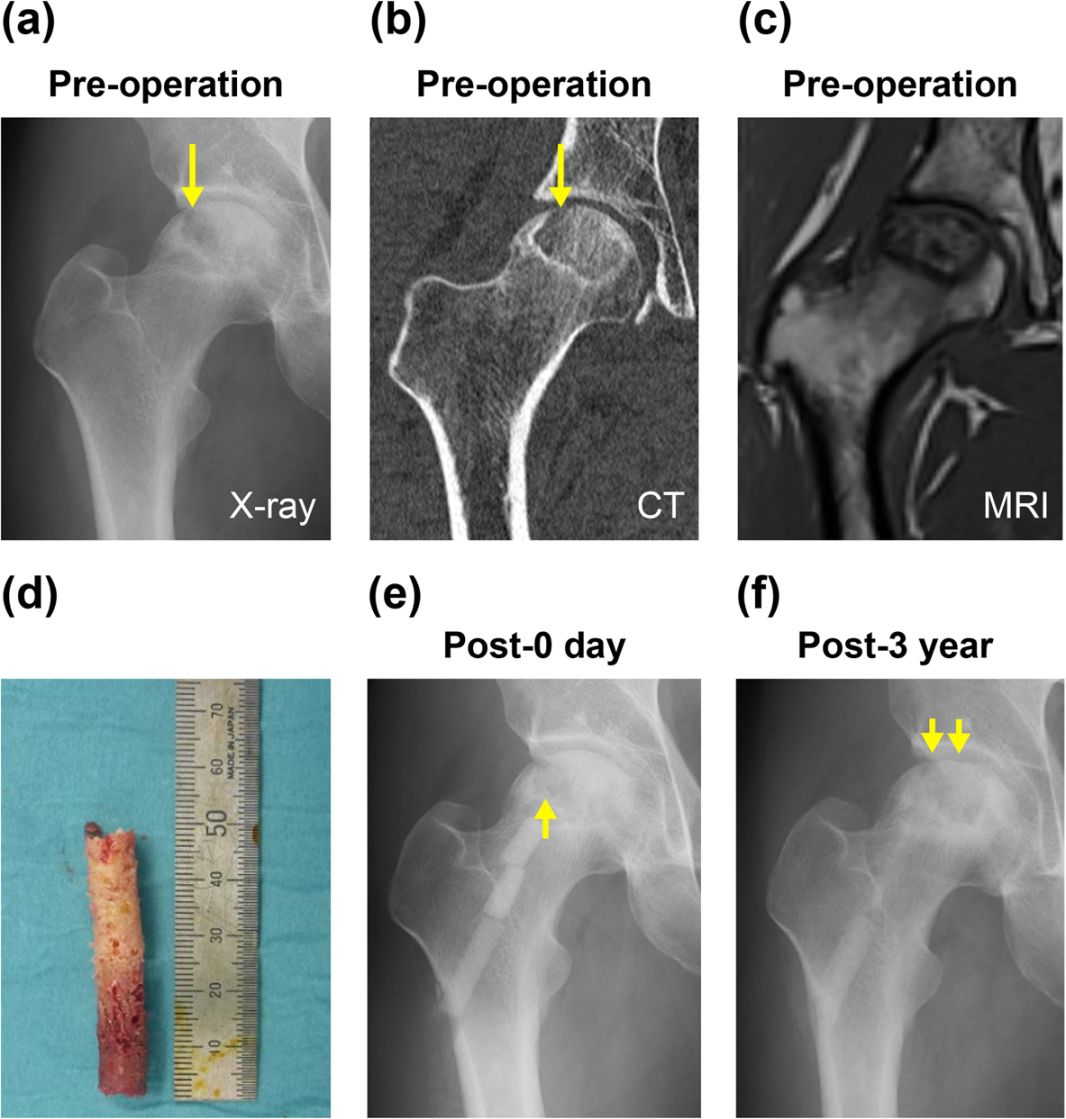
Representative pre- and postoperative radiological changes and an intraoperative photo (case 11). **a** Preoperative X-ray showing the presence of a demarcation line. **b** CT image showing a crack and bone absorption in the weight-bearing surface. **c** Preoperative coronal T1-weighted MRI showing a typical band pattern. **d** Intraoperative photograph of a cylindrical bone removed by CD. **e** Postoperative X-ray showing bone filling in the osteonecrotic area. **f** X-ray at 3 years showing radiological progression with flattening. The artificial bone in the femoral diaphysis is gradually resorbed. Downward yellow arrows indicate flattening or a subchondral crack, and the upward yellow arrow indicates femoral head spherification by IBG. CT, computed tomography; MRI, magnetic resonance imaging; IBG, impaction bone graft; CD, core decompression

**Fig. 4:**
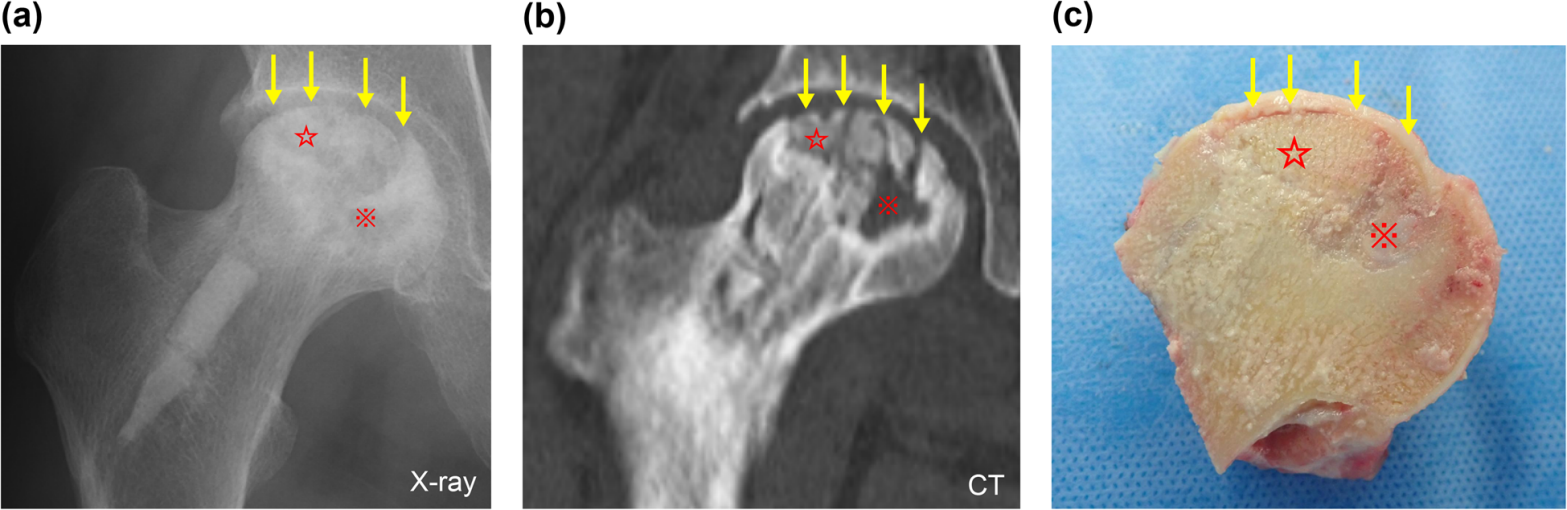
Radiographic images and a cross-sectional view of the explanted femoral head (case 20). Downward yellow arrows indicate flattening or a subchondral crack, red stars indicate the IBG site, and the red asterisk indicates the bone cyst area on the medial side of the femoral head. The graft bone remained, but the subchondral bone was flattened and detached from the cartilage. CT, computed tomography; IBG, impaction bone graft

The mean observation period was 36 months (range 29–42 months). The average surgery time was 54.5±21.6 min and tended to be longer in earlier stages of ONFH. The average blood loss was 42.7±83.0 g. At follow-up, 7 patients (35%) were converted to THA; the conversion was as trouble-free as normal primary THA. The survival rate with conversion to THA as the endpoint was 65% at 3 years postoperatively. Figure 2 shows results of Kaplan–Meier survival analysis with conversion to THA as an endpoint. The average time to THA was 17 months postoperatively (range 12–29 months). Radiological progression was observed in 12 patients (60%), including the 7 patients converted to THA, while 8 patients showed no stage change since surgery. Two patients (cases 5 and 7) underwent simultaneous surgery with unilateral THA and unilateral IBG surgery. One patient (case 11) showed femoral head collapse and osteosclerosis in the weight-bearing area, but the clinical course was good (Fig. 3). In contrast, case 20 showed bone resorption and bone cysts within the femoral head, leading to collapse and needing conversion to THA (Fig. 4). In all patients but excluded those who underwent THA, clinical scores significantly improved 3 years postoperatively (UCLA activity rating scale, 5.2 [*P* = 0.014]; HHS, 76.5 points [*P* = 0.005]) compared to preoperative scores (UCLA activity rating scale, 3.7; HHS, 57.6 points) (Table 2). At the latest follow-up, no patients scheduled THA.

**Table 2:** Changes in clinical outcomes of HHS and UCLA activity rating scale

Score (range)	Baseline	6 months	12 months	18 months	24 months	30 months	36 months	***P***-value
Number of patients assessed	20	20	20	15	14	14	6	
HHS (0–100)	57.6 ± 16.4	83.1 ± 11.7	78.7 ± 15.1	79.5 ± 14.4	79.4 ± 14.8	78.2 ± 16.3	76.5 ± 18.2	= 0.005^†^
	49.8 to 65.3	77.6 to 88.6	71.6 to 85.8	72.1 to 87.0	71.2 to 87.7	69.1 to 87.2	61.2 to 91.7	
	60.5 (21–83)	84.5 (56–96)	79.5 (52–100)	79.0 (52–10)	77.0 (52–100)	77.0 (42–100)	80.0 (42–96)	
UCLA scale (1–10)	3.7 ± 1.4	5.2 ± 1.0	5.3 ± 1.1	5.3 ± 0.9	5.5 ± 0.9	5.3 ± 0.9	5.2 ± 0.9	= 0.014^‡^
	3.0 to 4.3	4.7 to 5.6	4.7 to 5.9	4.8 to 5.8	5.0 to 6.0	4.8 to 5.9	4.4 to 6.1	
	3 (2–8)	5 (3–7)	5 (3–8)	5 (4–7)	5 (4–7)	5 (4–7)	5 (4–7)	

Data of the upper row are expressed as mean ± SD; middle row, 95% CI; and bottom row, median (range). The *P*-value was calculated for the effect of time in a repeated-measures linear mixed-effects model. The HHS ranged from 0 to 100.0, with a high positive value indicating a more functional hip joint. The UCLA activity rating scores ranged from 1 to 10, with 10 indicating “regularly participate in impact sports” and 1 indicating “wholly inactive, cannot leave residence”

*HHS* Harris hip score, *UCLA* University of California, Los Angeles, *SD* standard deviation, *CI* confidence interval

## Discussion

The hallmark of the percutaneous autologous bone graft surgical technique is IBG obtained by CD and reconstruction of the femoral head sphericity. Landgraeber et al. [17] used the same technique with a calcium sulfate (CaSO_4_)–calcium phosphate (CaPO_4_) composite bone graft substitute. They reported a survival rate of 75.9% (22/29) for precollapse ONFH at 2.5 years postoperatively.

Distinguishing the preoperative stage with or without femoral head collapse, Table 3 compares the results of this study to other surgical methods. The joint preservation rates of different CD-based surgical treatment options range from 13.0 to 92.8% in postcollapse ONFH [18–24]. The short-term results of this study do not compare favorably with other treatments. There are several possible reasons. First, the quality of bones collected by CD was variable. Although not assessed the volume of the collected bone intraoperatively, the amount of bone volume might be small for IBG. Cancellous bone quality is unpredictable preoperatively, and additional autogenous bone collection from the iliac bone should be considered. Surgery with only artificial bone grafting has poor outcomes. Liu et al. [24] implanted beta-tricalcium phosphate (β-TCP) in 23 advanced ONFH patients, of whom 20 (87%) were converted to THA at 5 years postoperatively. Allograft might still be better than artificial bone for ONFH, although its efficacy should be tested.

**Table 3:** Comparison of CD-based surgery and other NVG surgery outcomes for pre- and postcollapse-stage ONFH

First author/year	Surgical procedure	Mean age at surgery (years)	Mean follow-up (years)	Number of precollapse ONFH (hips)	Hip survivorship/endpoint	Number of postcollapse ONFH (hips)	Hip survivorship/endpoint
Seyler [18]/2008	Light bulb/NVG/rhBMP-7 (3.5 mg)	35	3	22	81.8% (18/22) Collapse	17	70.5% (12/17) THA
Wang [19]/2010	Light bulb/DBM, rhBMP	32.3	2.1	67	92.5% (62/67) THA	71	88.7% (63/71) THA
Wang [19]/2010	CD/BMMNC	37.5	2.3	50	78% (39/50) Radiological progression	9	66.6% (6/9) Radiological progression
Zhang [20]/2013	Light bulb/BG	31.3	Minimum 2	71	85.4% Collapse	14	NA
Lim [21]/2013	Multiple CD/Stem cell	36.3	5	79	62.7% (64/102) Surgery or HHS < 75	49	42.1% (24/57) Surgery or HHS < 75
Lim [21]/2013	Multiple CD	34.4	5	23		8	
Feng [22]/2019	CD/iliac BG/VGTF	33.2	9.7	None	NA	84	92.8% (78/84) THA
Feng [22]/2019	CD/iliac BG	32.8	9.8	None	NA	51	78.4% (40/51) THA
Tomaru [23]/2019	CD/BMMNC	42.2	12	43	51.1% (22/43) Collapse	37	43.2% (16/37) THA
Liu [24]/2020	CD/β-TCP	36	5	25	45.8% (11/24) THA	22	13.0% (3/23) THA
Present study/2020	CD/BG/HA, β-TCP	40.1	3	1	100% (1/1) Collapse	19	63.1% (12/19) THA

Hip survivorship was joint preservation rate with the bottom row as an endpoint

*NVG*, nonvascularized graft; *DBM*, demineralized bone matrix; *rh*, recombinant human; *BMP*, bone morphogenetic protein; *CD*, core decompression; *BMMNC*, bone marrow mononuclear cell; *BG*, bone graft; *VGTF*, vascularized greater trochanter flap; *β-TCP*, beta-tricalcium phosphate; *HA*, hydroxyapatite; *THA*, total hip arthroplasty; *NA*, not applicable; *HHS*, Harris hip score

Second, only autologous bone grafting might be insufficient. In the past 20 years, regenerative medicine has been developed for early-stage ONFH. The use of cells and growth factors has shown good results. Regenerative medicine can be applied to advanced cases, too. Wang et al. performed bone grafting by the light-bulb method combined with demineralized bone matrix with recombinant human bone morphogenetic protein (rhBMP) in advanced ONFH and reported an 88.7% (63/71) survival rate at 2 years postoperatively [19]. We have also reported good results of regenerative therapy for early-stage ONFH with recombinant human fibroblast growth factor-2 (rhFGF-2) [25]. Studies have reported low efficacy of cell therapy in advanced ONFH patients (Table 3). Tomaru et al. [23] reported a 43.2% (16/37) survival rate at 12 years postoperatively using bone marrow mononuclear cell (BMMNC) transplantation for advanced ONFH [23]. Wang et al. also reported a 66.6% (6/9) survival rate with BMMNC therapy at 2.3 years postoperatively [19]. This may be because glucocorticoid-induced ONFH patients have decreased angiogenic and increased apoptotic activity [26, 27]. Therefore, for the patients with glucocorticoid-induced ONFH, adjunct growth factors such as rhBMP might be more useful than the BMMNC therapy.

Third, the timing of weight bearing in postoperative rehabilitation is an issue. One-third PWB gait training started at 3 weeks postoperatively might be too early. Other techniques have postoperative weight-bearing restrictions for 1 or 2 months. It is necessary to establish a system to determine when gait training can begin.

Fourth, bilateral ONFH is difficult to treat. The bilateral ONFH incidence is as high as 75% [28]. A multicenter study reported that of 310 ONFH patients (505 hips), 62.9% (195/310) had bilateral ONFH [11]. While there is no definite surgical plan, ONFH progresses and eventually both sides might need THA. There is no consensus on management for different stages of bilateral ONFH.

Fifth is the mode of femoral head collapse related to the postoperative clinical score. The relationship between the mode of femoral head collapse and symptoms should be considered. Ito et al. [29] reported that sclerotic changes around ONFH show trabecular hypertrophy, slow bone resorption progression, and have a good clinical course. It is necessary to establish a method of intentionally leading the mode of collapse to the former, in which sclerotic change with minimal collapse of the femoral head will not cause further collapsing.

Finally, the increase in the number of ONFH surgeries and THA should be considered. A 20% sample of US hospitals had 3570 ONFH surgeries in 1992 and 6400 in 2008 [7]. This increase is accounted for by THA and hip resurfacing, with > 90% of surgeries being performed with artificial prostheses. Long-term THA outcomes for ONFH have been improving [7, 8]. Therefore, THA is readily selected as the final surgical option for advanced ONFH. However, if THA is selected as the final therapeutic solution in ONFH patients who underwent osteotomies with metal plates [9] or CD with tantalum rods [30], conversion to THA is difficult [1–4] and therefore on the decline [2, 6, 7]. We believe that for joint-preserving surgery, conversion to THA should be performed without difficulty.

This study has some limitations that must be noted. This was a retrospective study, with a small number of patients. The postoperative radiological assessment was used by X-ray for evaluating ONFH stage. But the results of the postoperative CT or MRI analysis are needed to evaluate bone regeneration on a radiological base. Further well-designed, high quality studies are required for future research.

## Conclusions

We developed and performed percutaneous IBG surgery for advanced ONFH. Although the surgical technique was simple and acceptable, the short-term results were not satisfactory. The clinical indications for this method will require caution. There are issues to be remedied such as the use of additional autologous bone, adjunctive regenerative therapy, and improvement of the rehabilitation therapy.

## Data Availability

All data generated or analyzed during this study are included in this published article.

## Abbreviations

β-TCP: Beta-tricalcium phosphate
BMMNC: Bone marrow mononuclear cell
BMP: Bone morphogenetic proteins
CaPO4: Calcium phosphate
CaSO4: Calcium sulfate
CD: Core decompression
CT: Computed tomography
FGF: Fibroblast growth factor
HHS: Harris hip scores
IBG: Impaction bone graft
JIC: Japanese Investigation Committee
ONFH: Osteonecrosis of the femoral head
PWB: Partial weight bearing
rh: Recombinant human
THA: Total hip arthroplasty
UCLA: University of California, Los Angeles
US: United States

## References

[1] Petek D, Hannouche D, Suva D (2019). Osteonecrosis of the femoral head: pathophysiology and current concepts of treatment. EFORT Open Rev..

[2] Mont MA, Salem HS, Piuzzi NS, Goodman SB, Jones LC (2020). Nontraumatic osteonecrosis of the femoral head: where do we stand today?: a 5-year update. J Bone Joint Surg Am..

[3] Moya-Angeler J, Gianakos AL, Villa JC, Ni A, Lane JM (2015). Current concepts on osteonecrosis of the femoral head. World J Orthop..

[4] Chughtai M, Piuzzi NS, Khlopas A, Jones LC, Goodman SB, Mont MA (2017). An evidence-based guide to the treatment of osteonecrosis of the femoral head. Bone Joint J..

[5] Atilla B, Bakırcıoğlu S, Shope AJ, Parvızı J (2020). Joint-preserving procedures for osteonecrosis of the femoral head. EFORT Open Rev..

[6] Kuroda Y, Matsuda S, Akiyama H (2016). Joint-preserving regenerative therapy for patients with early-stage osteonecrosis of the femoral head. Inflamm Regener..

[7] Johannson HR, Zywiel MG, Marker DR, Jones LC, McGrath MS, Mont MA (2011). Osteonecrosis is not a predictor of poor outcome in primary total hip arthroplasty: a systematic literature review. Int Orthop..

[8] Yang S, Halim AY, Werner BC, Gwathmey FW, Cui Q (2015). Does osteonecrosis of the femoral head increase surgical and medical complication rates after total hip arthroplasty? A comprehensive analysis in the United States. Hip Int..

[9] Nishio A, Sugioka Y (1971). A new technique of the varus osteotomy at the upper end of the femur. Orthop Traumatol..

[10] Phemister DB (1949). Treatment of the necrotic head of the femur in adults. J Bone Joint Surg Am..

[11] Ficat RP (1985). Idiopathic bone necrosis of the femoral head. Early diagnosis and treatment. J Bone Joint Surg Br..

[12] Kuroda Y, Tanaka T, Miyagawa T, Kawai T, Goto K, Tanaka S (2019). Classification of osteonecrosis of the femoral head: who should have surgery?. Bone Joint Res..

[13] Sugano N, Atsumi T, Ohzono K, Kubo T, Hotokebuchi T, Takaoka K (2002). The. 2001 revised criteria for diagnosis, classification, and staging of idiopathic osteonecrosis of the femoral head. J Orthop Sci..

[14] Amstutz HC, Thomas BJ, Jinnah R, Kim W, Grogan T, Yale C (1984). Treatment of primary osteoarthritis of the hip. A comparison of total joint and surface replacement arthroplasty. J Bone Joint Surg Am..

[15] Harris WH (1969). Traumatic arthritis of the hip after dislocation and acetabular fractures: treatment by mold arthroplasty. An end-result study using a new method of result evaluation. J Bone Joint Surg Am..

[16] Cnaan A, Laird NM, Slasor P (1997). Using the general linear mixed model to analyse unbalanced repeated measures and longitudinal data. Stat Med..

[17] Landgraeber S, Warwas S, Claßen T, Jäger M (2017). Modifications to advanced core decompression for treatment of avascular necrosis of the femoral head. BMC Musculoskelet Disord..

[18] Seyler TM, Marker DR, Ulrich SD, Fatscher T, Mont MA (2008). Nonvascularized bone grafting defers joint arthroplasty in hip osteonecrosis. Clin Orthop Relat Res..

[19] Wang BL, Sun W, Shi ZC, Zhang NF, Yue DB, Guo WS (2010). Treatment of nontraumatic osteonecrosis of the femoral head using bone impaction grafting through a femoral neck window. Int Orthop..

[20] Zhang HJ, Liu YW, Du ZQ, Guo H, Fan KJ, Liang GH (2013). Therapeutic effect of minimally invasive decompression combined with impaction bone grafting on osteonecrosis of the femoral head. Eur J Orthop Surg Traumatol..

[21] Lim YW, Kim YS, Lee JW, Kwon SY (2013). Stem cell implantation for osteonecrosis of the femoral head. Exp Mol Med..

[22] Feng W, Chen J, Wu K, Lu L, Deng P, Ye P (2019). A comparative study of cortico-cancellous iliac bone graft with or without the combination of vascularized greater trochanter flap for the management of femoral head osteonecrosis: a minimum 6 years follow-up. BMC Musculoskelet Disord..

[23] Tomaru Y, Yoshioka T, Sugaya H, Kumagai H, Hyodo K, Aoto K (2019). Ten-year results of concentrated autologous bone marrow aspirate transplantation for osteonecrosis of the femoral head: a retrospective study. BMC Musculoskelet Disord..

[24] Liu P, Mu XH, Yu HC, Guan JL, Liu ZH, Wang WG (2020). High failure rate after beta-tricalcium phosphate grafting for the treatment of femoral head osteonecrosis: a retrospective analysis. BMC Musculoskelet Disord..

[25] Kuroda Y, Asada R, So K, Yonezawa A, Nankaku M, Mukai K (2016). A pilot study of regenerative therapy using controlled release of rhFGF-2 for patients with pre-collapse osteonecrosis of the femoral head. Int Orthop..

[26] Yu H, Liu P, Zuo W, Sun X, Liu H, Lu F (2020). Decreased angiogenic and increased apoptotic activities of bone microvascular endothelial cells in patients with glucocorticoid-induced osteonecrosis of the femoral head. BMC Musculoskelet Disord..

[27] Houdek MT, Wyles CC, Packard BD, Terzic A, Behfar A, Sierra RJ (2016). Decreased osteogenic activity of mesenchymal stem cells in patients with corticosteroid-induced osteonecrosis of the femoral head. J Arthroplasty..

[28] Nam KW, Kim YL, Yoo JJ, Koo KH, Yoon KS, Kim HJ (2008). Fate of untreated asymptomatic osteonecrosis of the femoral head. J Bone Joint Surg..

[29] Ito H, Matsuno T, Omizu N, Aoki Y, Minami A (2003). Mid-term prognosis of non-traumatic osteonecrosis of the femoral head. J Bone Joint Surg..

[30] Cheng Q, Tang JL, Gu JJ, Guo KJ, Guo WS, Wang BL (2018). Total hip arthroplasty following failure of tantalum rod implantation for osteonecrosis of the femoral head with 5- to 10-year follow-up. BMC Musculoskelet Disord..

